# Psychedelics and workplace harm

**DOI:** 10.3389/fpsyt.2023.1186541

**Published:** 2023-06-16

**Authors:** Sean Matthew Viña, Amanda Layne Stephens

**Affiliations:** ^1^University of the Incarnate Word, San Antonio, TX, United States; ^2^St. Mary’s University School of Law, San Antonio, TX, United States

**Keywords:** health, mental health, distress, work hour limitation, psychedelics

## Abstract

This study aims to understand the relationship between Lifetime Classic Psychedelic Use (LCPU), employment status, and weekly work hours on levels of psychological distress. The data used for this analysis is pooled from the National Survey of Drug Use and Health (NSDUH) from 2008–2019 and includes a sample size of 484,732 individuals. The findings suggest that LCPU and being employed are independently associated with better health. Additionally, the results indicate that psychedelic use is associated with lower distress for those who are employed, volunteer, retired, or permanently disabled. However, those who are unemployed, full-time students, or homemakers may experience higher levels of distress with psychedelic use. Interestingly, the analysis also suggests that those who use psychedelics are working longer hours per week before experiencing an increase in stress. Overall, the study suggests that psychedelics are not likely to have a negative impact on employment outcomes.

## 1. Introduction

The use of psychedelics is rapidly increasing, according to the latest wave of the Monitoring the Future (MTF) panel study. The study found that recreational use of hallucinogens, including LSD, psilocybin mushrooms, and other psychedelic substances, increased from 5 to 9% among college students between 2019 and 2020 (1). There are even reports of a rise in psychedelic use among middle-class Americans, including white mothers (2).

The increase in the use of psychedelics can be attributed to two factors. Firstly, research has demonstrated that psychedelics can have a positive impact on health, including reducing depression (3, 4), anxiety (5, 6), suicide (7), PTSD (8–10), drug dependency (11–13), cardiovascular health (14–16), and negative social behaviors such as violence and larceny (17–19). And secondly, there have been significant legal changes regarding the legality of psychedelics which is particularly attributed trend in mental health professionals increasingly accepting their use (20–22). Because of the increasing commodification of psychedelics, their use is becoming both more available and legitimate to new and wider populations of potential users.

Despite these social and political trends, the societal impact of psychedelic drug use is not well-studied, including its effects on the workplace. Two important questions need to be explored: first, how will the increase in psychedelic use affect the workplace? Many workplaces are now recognizing the importance of promoting a sustainable lifestyle through education, resources, and activities. As social acceptance of psychedelic use grows, it’s crucial to understand how these drugs could affect both the workplace and employee health. Considering evidence that finds psychedelic use is associated with better health and behaviors, it is likely that psychedelic use will not negatively impact the workplace. On the other hand, it is possible that psychedelics could follow patterns similar to other drugs and alcohol, which are associated with negative social outcomes and high rates of violence (23–26).

And secondly, does employment status impact the effectiveness of psychedelics from a clinical perspective? The role of sociocultural conditions in shaping the set-and-setting of psychedelic experiences has been suggested as a crucial factor (27–34). Setting refers to the physical and social environment where psychedelics are consumed. In contrast, set “refers to the internal conditions of the person using the psychedelics, including factors such as mood, attitudes, preparation, personal history, personality, expectations, motivations for using, and beliefs about themselves and the use of drugs” [(35), p. 52]. Accordingly, “the therapeutic action of psychedelics is fundamentally reliant on context – both in the psychological and environmental sense” [(27), p. 725]. Set-and-setting could be hypothesized as sitting on a spectrum from the most optimal to the most compromised. Those with the most optimal set-and-setting have the conditions (i.e., psychological, biological, and social) that create the best sustained health outcomes. Conversely, those with a compromised set-and-setting have less of the necessary conditions to create optimal or sustained health outcomes associated with psychedelic use. All things being equal, any disparate outcomes associated with psychedelics suggests one has a better set-and-setting than another although the direct cause of the disparity may be unknown. For instance, several studies have found the most fulfilling psychedelic experiences were those that drew upon positive personal experiences or relationships (36).

Overall, cultural set-and-setting suggests that employment status will be a major facilitator of different psychedelic health outcomes for a few reasons. First, drawing on theory of fundamental causality, those who are employed tend to have higher levels of key resources that impact health and wellbeing including knowledge, money, power, prestige, and beneficial social (37, 38). Moreover, those who are chronically unemployed are at higher risk of all the top 10 leading causes of death in America, which include heart disease, cancer, COVID, and accidents (39). Those who are unemployed also have higher rates of stress; are more likely to use harmful substances, including tobacco and alcohol (40); and are less likely to have healthy diets or exercise (41). The magnitude of illness and stress among those who are unemployed illustrates why they will have a compromised set-and-setting and thus benefit less from psychedelic use. Those who are unemployed have higher rates of mental illness, distress, and anxiety, which may make it harder to get into a positive mindset before a psychedelic trip. Those who are unemployed may also have fewer positive experiences to draw upon to interpret the trip, or the positive effects of psychedelics will be eliminated over time faster because of chronic stress.

This study investigates the associations between employment status, weekly work hours, and lifetime classic psychedelic use (LCPU) on psychological distress in a nationally representative sample of the United States. The National Survey of Drug Use from 2008 to 2019 was used, which included 484,732 participants. The results show that psychedelic use is associated with lower distress for those who are employed full or part-time, volunteer, retired, or permanently disabled. However, psychedelic use is associated with higher distress for those who are unemployed, full-time students, or homemakers. Additionally, a quadratic regression suggests that those who have used psychedelics are working substantially more hours per week before stress increases (about 3–4 times longer), compared to those who have not used psychedelics. Overall, the results suggest that psychedelics may not be harmful to the workplace and may even promote better health and a sustainable lifestyle. All quotes and materials used in this study are cited appropriately.

## 2. Data and methods

This study analyzed data from the National Survey of Drug Use and Health (NSDUH) from 2008 to 2019. The NSDUH is an annual survey conducted in all 50 states and the District of Columbia to measure substance use and mental health issues in the United States. The data was weighted to reflect the civilian noninstitutionalized population and included responses from 674,521 individuals. Descriptive statistics for dependent, independent, and control variables are shown in Table 1. All variables were derived from publicly available data, and full sampling techniques can be found on the NSDUH website.^1^

**Table 1:** Descriptive statistics for dependent variables, independent variables, and controls (2008–2019) (weighted).

	Mean	SD	*n*	% / min-max
Dependent variable				
Psychological distress in last month (K6)	9.58	6.08	161,573	0–24
LCPU			85,451	12.67
Independent variables				
Employment status				
Employed full-time			229,207	47.52
Employed part-time			56,697	11.75
Unemployed			41,182	8.54
Volunteer			22,422	4.65
Disabled			24,818	5.15
Homemaker			20,680	4.29
Student			10,746	2.23
Retired			76,597	15.88
Hours worked last week	23.88	21.36	455,547	0–61
Control variables				
Women			250,941	51.77
Age	8.67	2.31	484,732	1–11
Race				
White			318,260	65.66
Black			56,783	11.71
Native American			2,543	0.52
Hawaiian			1,735	0.36
Asian			24,929	5.14
Multi-racial			7,188	1.48
Hispanic			73,290	15.12
Marital status				
Married			254,500	47.50
Single, never married			134,006	27.65
Widowed			28,907	5.96
Divorced/separated			67,316	13.89
Educational attainment				
Less than high school			66,274	13.67
High school			34,881	27.83
Some college			136,684	28.20
College degree or higher			146,891	30.30
Annual household income	4.92	2.60	484,732	1–7
Religious attendance	1.89	1.88	480,882	0–5
Religious salience	4.92	2.60	472,653	0–9
Mental health treatment			69,649	14.45
Age of first alcohol use	1.61	0.47	484,732	1–5
Lifetime drug use				
Tobacco			277,750	57.30
Cocaine			78,221	16.14
Stimulants			49,164	10.14
Sedatives			40,022	8.26
Tranquilizer			79,691	16.44
Inhalants			42,850	8.85
Pain relievers			174,373	35.97
Heroine			9,527	1.97
Marijuana			225,160	46.45
PCP			12,642	2.61
MDMA/ecstasy			34,224	7.06
Self-reported risky behavior	1.61	0.74	483,056	1–4

2008–2019 National Survey of Drug Use and Health, *n* = 484,732.

### 2.1. Dependent variables

The respondents used the Kessler Psychological Distress Scale (K6) to report their level of distress in the past month (42, 43). Using a 5-point Likert scale, participants indicated how often they experienced six different feelings or experiences in the past 30 days. These included feeling “nervous,” “hopeless,” “restless or fidgety,” “so depressed that nothing could cheer you up,” “that everything was an effort,” and “worthless.” The resulting variable for psychological distress in the past month was created by adding all measures into one scale, ranging from 0–24. Higher scores indicate more distress.

#### 2.1.1. Independent variables

The first independent variable is classic psychedelic use, a subclass of psychedelics that has little toxicity (44–46). The three main classes of classic psychedelics—including tryptamines, lysergamides, and phenethylamines—are distinguished by unique chemical structures and neurochemical mechanisms (47). Classic psychedelics include N-dimethyltryptamine (DMT), the DMT-containing admixture ayahuasca, psilocybin, lysergic acid diethylamide (LSD), mescaline, and the mescaline-containing cacti peyote. Respondents reported if they had ever used, even once, the following drugs: DMT, ayahuasca, LSD, mescaline, peyote, or psilocybin. Consistent with previous research (14–16, 48), the six variables were used to create two different variables for the analysis. A dummy variable was created indicating any lifetime classic psychedelic use (LCPU) (yes vs. no). The dummy variable was used to compare the mean differences of all variables by LCPU status (Table 2).

**Table 2:** Means differences of key variables and control variables by LCPU (Weighted).

	Mean		Mean difference ^a^	
	No LCPU	LCPU	No LCPU (−) LCPU	
Psychological distress in past month (K6)	9.26	10.91	−1.62	***
	(0.027)	(0.049)	(0.051)	
Employment status				
Employed full-time	0.46	0.56	−0.10	***
Employed part-time	0.11	0.12	−0.01	***
Unemployed	0.08	0.08	−0.01	*
Volunteer	0.04	0.04	0.00	
Disabled	0.04	0.06	−0.01	***
Homemaker	0.04	0.02	0.01	***
Student	0.02	0.01	0.01	***
Retired	0.17	0.05	0.11	***
Hours worked last week	23.17	28.34	−5.16	***
	(0.063)	(0.149)	(0.154)	
Age	8.68	8.55	0.13	***
	(0.006)	(0.011)	(0.011)	
Women	0.54	0.37	0.16	***
Race				
White	0.62	0.82	−0.19	***
Black	0.12	0.03	−0.09	***
Native American	0.00	0.01	−0.00	***
Hawaiian	0.00	0.00	−0.00	**
Asian	0.05	0.01	0.04	***
Multi-racial	0.01	0.02	−0.01	***
Hispanic	0.16	0.08	0.07	***
Marital status				
Married	0.53	0.45	0.07	***
Single, never married	0.26	33	−0.06	***
Widowed	0.06	0.02	0.04	***
Divorced/separated	0.13	0.18	−0.05	***
Educational attainment				
Less than high school	0.14	0.09	−0.04	***
High school	0.28	0.25	−0.02	***
Some college	0.27	0.33	−0.05	***
College or higher	0.30	0.31	−0.01	***
Annual household income	4.94	5.10	−0.16	***
	(0.007)	(0.014)	(0.014)	
Religious attendance	2.00	1.20	0.79	***
	(0.005)	(0.011)	(0.012)	
Religious salience	5.11	3.57	1.36	***
	(0.006)	(0.016)	(0.017)	
	0.12	0.23	−0.10	***
Age of first alcohol use	3.01	2.00	1.01	***
	(0.002)	(0.004)	(0.005)	
Lifetime drug use				
Tobacco	0.52	0.89	−0.37	***
Cocaine	0.07	0.70	−0.63	***
Stimulants	0.06	0.35	−0.29	***
Sedatives	0.06	0.22	−0.16	***
Tranquilizer	0.12	0.42	−0.29	***
Inhalants	0.04	0.38	−0.34	***
Pain relievers	0.31	0.62	−0.30	***
Heroine	0.00	0.11	−0.11	***
Marijuana	0.38	0.98	−0.59	***
PCP	0.00	0.16	−0.16	***
MDMA/ecstasy	0.02	0.36	−0.34	***
Self-reported risky behavior	1.55	2.00	−0.45	***
	(0.001)	(0.005)	(0.005)	

2008–2019 National Survey of Drug Use and Health, *n* = 484,732.

a Calculated in STATA with LINCOM (Linear and nonlinear combination) post estimation commands.

^b^Standard deviations in parentheses.

^†^*p* < 0.10, ^*^p < 0.05, ^**^p < 0.01, ^***^p < 0.001 (two-tailed).

Employment status is a variable with eight categories: (1) part-time employment, (2) unemployment, (3) volunteering, (4) disability, (5) homemaking, (6) student, (7) retirement, and (8) full-time employment, which is the reference category. Respondents were asked to report the number of hours they work per week, which is a continuous variable ranging from zero to 60 or more hours.

### 2.2. Control variables

This study replicates other studies by including the same control variables related to sociodemographic, drug use, and risky behavior (14–16, 49). Sociodemographic control variables include two continuous variables, age (18, 19, 20, 21, 22–23, 24–25, 25–29, 30–34, 35–49, 50–64, and 65+) and annual household income (less than $10,000, $10,000–$19,999, $20,000–$29,999, $30,000–$39,999, $40,000–$49,999, $50,000–$74,999, and $75,000 or more). The analysis includes multiple dummy variables, gender (women versus men), race/ethnicity (non-Hispanic African American, non-Hispanic Native American/Alaska Native, non-Hispanic Native Hawaiian/Pacific Islander, non-Hispanic Asian, non-Hispanic more than one race, Hispanic, and non-Hispanic white, serving as the reference category), and educational attainment (high school degree, some college, college degree or higher, and less than a high school degree, serving as the reference category), and Marital (single, never married, widowed, divorced/separated, and married, serving as the reference category). There are two continuous variables measuring religiosity. First, religious attendance is a continuous measure of how often a person attended religious services in the last year with the following option, (0=) 0 ties, (1=) 1 to 2 times, (2=) 3–5 times, (3=) 6 to 24 times, (4=) 25 to 52 times, and (5=) more than 52 times. Respondents also responded how much they agree to the following three statements: (1) my religious beliefs are very important, (2) my religious beliefs influence life, and (3) it’s important that I associate with religious people, which were summed to create a measure of religiosity which range from 1 to 4 (Cronbach’s alpha = 0.84). Binary control variables for lifetime drug use include use of cocaine; marijuana use; 3,4-methylenedioxymethamphetamine (MDMA/ecstasy); phencyclidine (PCP); inhalants; other stimulants; sedatives; pain relievers; and tobacco (smokeless tobacco, pipe tobacco, cigar, and daily cigarette). The age of first alcohol use and risky behaviors are both continuous variables. Finally, the regression analysis also controls for the year of the survey.

### 2.3. Analytic strategy

To address this study’s questions, we began by calculating the mean of each variable in the sample by LCPU. Then, we conducted a post-estimation LINCOM (non-linear combination) commands, which compute the statistical difference of two subpopulation means (50). We calculated the statistical mean difference of LCPU minus (−) No LCPU for dependent, independent, and control variables (Table 2). The analysis uses series of ordinary least square regression models to test the relationship between employment status, weekly work hours, LCPU, and psychological distress over the past month (Table 3). The first model predicts LCPU on psychological distress with all controls. Model 2 third model includes weekly work hours and a quadratic of work hours to account for a curvilinear relationship. Model 3 is the full model that includes both employment and weekly work hours. Model 5 includes an interaction between weekly work hours and LCPU. Finally, model 5 includes an interaction between employment status and LCPU. The analysis also ran regression post-estimation Wald to see measure the equality of coefficients between psychedelic use and nonuse for different employment statuses (e.g., LCPU vs. no LCPU among the disabled).

**Table 3:** Weighted multivariate ordinary least square regression predicting the level of psychological distress in the past month.

	Model 1	Model 2	Model 3	Model 4	Model 5
Independent variables					
LCPU	−0.1477^*^	−0.1378^*^	−0.1528^*^	0.1322	−0.3277^***^
	(0.0570)	(0.0605)	(0.0618)	(0.1028)	(0.0826)
Work hours		−0.0225^***^	−0.0062	−0.0048	−0.0066
		(0.0037)	(0.0085)	(0.0084)	(0.0085)
Work hours squared		0.0004^***^	0.0002	0.0002	0.0002
		(0.0001)	(0.0001)	(0.0001)	(0.0001)
Employment status					
Employed part-time			−0.0367	−0.0358	−0.0895
			(0.0837)	(0.0837)	(0.0844)
Unemployed			0.7506^***^	0.7414^***^	0.5974^**^
			(0.1764)	(0.1762)	(0.1855)
Volunteer			−1.2342	−1.2370	0.1239
			(1.5608)	(1.5684)	(1.2713)
Disabled			1.9509^***^	1.9367^***^	1.9672^***^
			(0.1934)	(0.1935)	(0.2039)
Homemaker			0.3938^*^	0.3978^*^	0.2703
			(0.1939)	(0.1937)	(0.1978)
Student			−0.7402^***^	−0.7328^***^	−0.8682^***^
			(0.1981)	(0.1978)	(0.2042)
Retired			−0.7694^***^	−0.7457^***^	−0.8350^***^
			(0.1819)	(0.1816)	(0.1849)
Interaction terms					
LCPU*					
Work Hours				−0.0106^***^	
				(0.0030)	
Employed part time					0.2579
					(0.1634)
Unemployed					0.7405^***^
					(0.2000)
Volunteer					−3.6624
					(2.6685)
Disabled					−0.0574
					(0.2300)
Homemaker					0.7350^*^
					(0.2835)
Student					0.7810^**^
					(0.2667)
Retired					0.5161
					(0.2787)
Constant	13.2429^***^	13.3978^***^	12.8359^***^	12.7944^***^	12.8948^***^
	(0.1842)	(0.1949)	(0.2627)	(0.2611)	(0.2631)
Observations	158,313	148,965	148,917	148,917	148,917
*R* ^2^	0.227	0.228	0.238	0.238	0.238

2008–2019 National Survey of Drug Use and Health, *n* = 484,732.

Standard errors in parentheses.

^*^ *p* < 0.05, ^**^ *p* < 0.01, ^***^ *p* < 0.001.

All models include controls for age, gender, race/ethnicity, religious attendance, religious salience, mental health treatment in the past year, lifetime drug use (cocaine, stimulants, sedatives, tranquilizers, heroine, pain killers, marijuana, PCP, ecstasy, inhalants, tobacco), age of first alcohol use, risky behaviors, and the survey year. Full models available in the Supplementary Table S1.

NSDUH created weights by adjusting the single-year weights by a scalar factor (i.e., the number of years of data used) so that the estimated number of individuals reported is representative of the national population. All analyses incorporate the sampling weights and complex study design provided by the NSDUH survey and conducted in STATA 17. Because the analysis includes weights, results are presented with presented with *p*-values and unstandardized coefficients; standardized effects including partial R-squared statistics or Cohen’s D cannot be reported. The analysis presented all pooled data from 2008–2019. Except for the addition of employment status and work hours, this study replicates previous studies and includes the same the same control variables within logistic regressions (7, 17, 18, 48, 51). All regression results are presented as we also follow those studies by pooling all available data in the NSDUH. Finally, as with previous research on psychedelics using the NSDUH, this study does not include controls for multiple comparisons (15, 16, 52). However, according to (53), a Bonferroni correction is not needed for this study because we meet the following requirements: (1) we do not require a single test of the universal null hypothesis, (2) we do not need to avoid a type I error, and (3) we have our study is driven by preplanned hypotheses on how employment status will affect the relationship between psychedelic and health.

## 3. Results

### 3.1. Descriptive statistics

Table 2 presents the mean difference of weighted descriptive statistics by LCPU status. Results indicate that who are employed full time were more likely to use LCPU than not, and they had the highest LCPU use (*p* < 0.001). Those who are employed part-time or disabled are slightly more likely to have used psychedelics (*p* < 0.001). Those who were unemployed were more likely to use psychedelics, but only at the 0.05 level. There was no association between being a volunteer and LCPU status. Students, homemakers, and those who are retired are less likely to have ever used psychedelics (*p* < 0.001). LCPU is associated with an average of five more hours of work each week compared to those who have never used psychedelics (*p* < 0.001). Finally, descriptive statistics align with previous research that finds psychedelic users are more likely to be men, white, native American, single, divorced, have a college education or higher, be wealthier, less religious, have uses any drug or started drinking earlier, and self-report more risky behaviors (*p* < 0.001).

### 3.2. Main effects of LCPU, employment status, and weekly work hours

Table 3 presents results from the weighted ordinary least square regression. Model 1 demonstrates that LCPU is associated with less psychological distress (b = −0.1477, *p* < 0.01), which remains significant in the full model when which includes both employment status and weekly work hours (b = −0.1528, *p* < 0.01). Model 2 reveals a statistically significant relationship between distress and work hours. Results indicate that each 1 hour increase in the number of hours worked is associated with less distress (b = −0.8378, *p* < 0.001), but the association is decreasing at a decreasing rate (b = 0.0004 *p* < 0.001) so that longer work hours per week become positively associated with higher distress. However, in the full model (model 3) that adds employment status, the association between work hours and distress disappears, which indicates that employment status may be more important for the relationship between psychedelic and health than work hours. Furthermore, compared to those who are employed full time, those who are unemployed (b = 0.7052, *p* < 0.001), disabled (b = 1.8924, *p* < 0.001), or homemakers (b = 0.3512, *p* < 0.001), have higher levels of stress. Compared to those who are full-time employed, those who are students (b = −0.7627, *p* < 0.001) or retired (b = −0.8378, *p* < 0.001) have less stress. There was no statistical difference in levels of distress between those who are employed full-time, part-time, or volunteer.

#### 3.2.1. Two-way interactions

**Figure 1:**
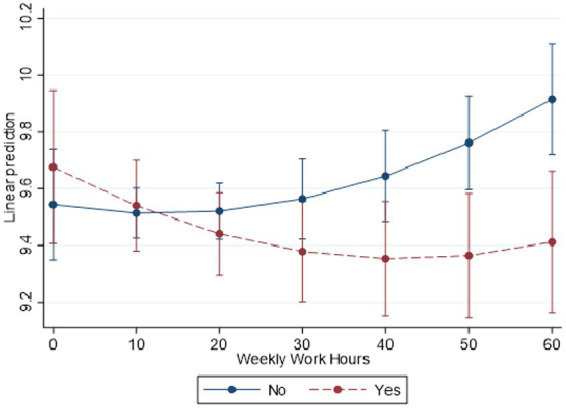
Predicted margins of LCPU*hours worked in past week with 95% CIs. National Survey of Health and Social Behaviors, 2008–2019. Based on Model 5, Table 3, multinomial OLS regression model predicting psychological distress in the past 30 days (k6).

Models 4 and 5 present two-way interactions between LCPU with employment status and weekly work hours, respectively. Model 4 shows that the negative curvilinear relationship between work hours and distress is amplified by LCPU (b = −0.0106, *p* < 0.001). Compared to those who have not used psychedelics, those who have used psychedelics are working about 3–4 times longer each week before distress increases (Figure 1). Model 5 shows that compared to those who are employed full-time, the negative association between LCPU and health is reduced for those who are unemployed (b = 0.7405, *p* < 0.001), homemakers (b = 0.7350, *p* < 0.05), and students (b = 0.7810, *p* < 0.01). Post estimation Wald-test indicated that while LCPU is associated with less stress for those who are employed full (*p* < 0.001) or volunteering (*p* < 0.05), LCPU is associated with higher levels of stress for those who are unemployed (*p* < 0.01), homemakers (p < 0.01), and students (*p* < 0.01). Psychedelic use was not significantly associated with a difference in distress or for those who were employed part-time or retired. These combined results suggest those who are employed receive the most benefits from psychedelics, and the association between work hours and stress may be positively impacted by LCPU.

### 3.3. Sensitivity analysis

Although this study replicates previous analyses, there may be a question of significance with such a large data set. Therefore, this study uses a sensitivity analysis by running the full analysis with only 2019 data. It was appropriate to systemically add one additional year and replicate the analysis; 2018–2019, 2017–2019, and then 2016–2019. Results were substantively identical, except that the interaction between LCPU and being unemployed was only significant at the 0.05 level (Model 5). The two-way interaction term between LCPU and homemaker or being a student were not significant (Model 5). Those interactions became significant at the 0.05 level with three pooled years of data. The interaction between LCPU and hourly work hours was significant at the 0.05 level (Model 6) with 2 years of pooled data. Lastly, a sensitivity analysis found that the interaction between LCPU and the quadratic of weekly work hours was not significant. Results are discussed.

## 4. Discussion

This study has addressed important gaps by testing the associations between employment status, the number of hours worked each week, and psychedelic use on distress. The potential interplay between economic forces and psychedelics remains understudied. This study addressed two main questions: (1) do psychedelics affect the workplace, specifically related to health? And (2) does employment status affect the efficacy of psychedelics on health? To address these questions, the analysis uses data from a nationally represented sample of Americans. Results demonstrate a complementary relationship between employment, LCPU, and distress. Specifically, those who are employed appear to gain the most benefits from psychedelic use while psychedelic users who are unemployed have higher levels of distress. Also, psychedelic use is associated with less distress in the workplace over a week of work, which suggests that psychedelic use may help promote better health and a sustainable lifestyle in the workplace.

These results need to be interpreted carefully. Some may wrongly conclude that psychedelics lead to less stress in the workplace, and consequently, psychedelics should be recommended by employers to increase productivity. These results only demonstrate an association, not causation. One possibility is that sustained health benefits found in clinical trials are being captured by this single measure of lifetime psychedelic use, which are then attenuated by employment status and work hours. Another possibility is that the association is spurious, specifically that those who use psychedelics are also more likely to have a healthy lifestyle and less stressful job. Scholars note that psychedelic exceptionalism—claims that psychedelics should be privileged for reform over more dangerous drugs like heroin and cocaine—is largely driven by the upper class who are at low risk for negative legal consequences for drug use (54). In other words, the results from this paper may be driven in part by unknown social and class privileges, not necessarily the drugs themselves. While future experimental studies can better parse out the causal mechanisms, the results still show that those who have chosen to use psychedelics are also experiencing less stress in the workplace, which lends support for the growing call to decriminalize psychedelics because they do not appear to be associated with societal harm.

Second, these results should be considered by researchers and counselors who are interested in the therapeutic aspects of psychedelics. Psychedelic-assisted therapies carefully prime individuals prior to the psychedelic trip, to create positive set-and-setting (10). These results suggest there may be limits to the efficacy of these drugs if people do not have ample economic resources outside of the clinic. Psychedelic therapeutic use alone may not be enough, especially considering those with severe mental illness who have the highest need for psychedelic care also have the highest unemployment rate. In addition to providing therapeutic care, there may be a need to connect patients with external economic and social resources to create sustained benefits of psychedelics post treatment.

## 5. Limitations and future directions

While this study reveals important associations between employment, work hours, distress, and psychedelics, it also has limitations. First, the primary limitation is data. It is possible that those who are unemployed or working long hours gain some benefit from psychedelics in the short term that are simply diminished over time. Longitudinal data that has the time of drug use would better indicate the decline in psychedelic efficacy. Apart from examining whether different job types (e.g., manual vs. office work) can account for differences on how psychedelics interact with employment and work patterns, it would be important to examine changes that individuals undergo before, during and after the period of their psychedelic use. Second, unmeasured endogenous factors could also be driving the association among jobs, psychedelics, and distress. We included a host of standard control sociodemographic control variables, but this is likely not an exhaustive list. In particular, common findings in the psychedelic clinical trial literature suggest other variables could affect outcomes, including personality traits, presence of peak experience, response to peak experience, and dosage. Most importantly, given the cross-sectional study design, the results cannot be used to make conclusive causal inferences especially because we do not know the motivation for use. Those who use psychedelics in clinical setting are likely doing so for health benefits while those who use it in a naturalistic setting will have many different motivations. For example, are individuals able to work much longer hours because of their psychedelic drug use when comparing their before and after psychedelics performances? Or could it be that these are individuals who would in any case work longer hours and use psychedelics, perhaps because their jobs allow for it in ways that others would not (e.g., office vs. manual, or IT vs. other jobs)? Future research should ask for more precise indicators of job types and motivation of psychedelic use, so we can understand how people are using psychedelics.

One may ask why research should consider employment status results from a population-level approach using a single lifetime use of classic psychedelics rather than those from clinical trials? First, these results represent how a population interacts with drugs in their everyday lives instead of in a controlled clinical environment. As psychedelics become more widely available, naturalistic use will rise faster than clinical treatment, especially in places where mental health treatment is severely underfunded.

Second and directly related, regardless of the motivation (clinical or recreational), psychedelics are associated with less distress and many other health benefits that are found in nationally represented samples. Population studies are worthwhile so that researchers can investigate whether this holds true among all groups including differently employed. Here, we found that positive outcomes did not exist for those who were unemployed. Ultimately, all people live in communities and social situations that directly impact the efficacy of psychedelics, even those used in a clinic, and we need to acknowledge the social situation. Therefore, even if unemployed people benefit within controlled clinical settings, whether they continue to enjoy those benefits in their everyday lived experiences remains another question.

## 6. Conclusion

These limitations notwithstanding, this study adds a critical new piece to the burgeoning research on psychedelics, health and wellbeing, and employment. It demonstrates how social conditions can affect the association between psychedelics and health. It also adds evidence to the growing literature that psychedelics may be beneficial to different parts of society.

## Data availability statement

Publicly available datasets were analyzed in this study. This data can be found here: https://www.datafiles.samhsa.gov/dataset/nsduh-2002-2019-ds0001-nsduh-2002-2019-ds0001.

## Ethics statement

The studies involving human participants were reviewed and approved by NA. This study uses publicly available secondary data. The patients/participants provided their written informed consent to participate in this study.

## Author contributions

SV is responsible for the theoretical framing on cultural set-and-setting, statistical analysis, and ensuring that the descriptions are accurate and agreed to by all authors. AS provided theoretical framing on occupational inequality and editing for the final paper. All authors contributed to the article and approved the submitted version.

## Conflict of interest

The authors declare that the research was conducted in the absence of any commercial or financial relationships that could be construed as a potential conflict of interest.

## Publisher’s note

All claims expressed in this article are solely those of the authors and do not necessarily represent those of their affiliated organizations, or those of the publisher, the editors and the reviewers. Any product that may be evaluated in this article, or claim that may be made by its manufacturer, is not guaranteed or endorsed by the publisher.

## Footnotes

^1^ https://nsduhweb.rti.org/respweb/about_nsduh.html
