# Supplementary material for: Psychedelics and workplace harm
Source: Front Psychiatry. 2023 Jun 16;14:1186541. doi: 10.3389/fpsyt.2023.1186541 (PMC10311554; doi:10.3389/fpsyt.2023.1186541)
Supplement: Supplementary file 1 [file Table_1.DOCX]

**Supplemental Tables**

| **Supplemental Table 1.** Weighted Multivariate Ordinary Least Square Regression Predicting the Level of Psychological Distress in the Past Month | | | | | | |
| --- | --- | --- | --- | --- | --- | --- |
|  | Model 1 | Model 2 | Model 3 | Model 4 | Model 5 | Model 6 |
| Independent Variables |  |  |  |  |  |  |
| LCPU | -0.1477^*^ | -0.1572^**^ | -0.1378^*^ | -0.1528^*^ | -0.3277^***^ | 0.1322 |
|  | (0.0570) | (0.0584) | (0.0605) | (0.0618) | (0.0826) | (0.1028) |
| Employment Status ^a^ |  |  |  |  |  |  |
| Employed Part -Time |  | -0.1180 |  | -0.0367 | -0.0895 | -0.0358 |
|  |  | (0.0661) |  | (0.0837) | (0.0844) | (0.0837) |
| Unemployed |  | 0.7052^***^ |  | 0.7506^***^ | 0.5974^**^ | 0.7414^***^ |
|  |  | (0.0784) |  | (0.1764) | (0.1855) | (0.1762) |
| Volunteer |  | 0.1124 |  | -1.2342 | 0.1239 | -1.2370 |
|  |  | (0.0954) |  | (1.5608) | (1.2713) | (1.5684) |
| Disabled |  | 1.8924^***^ |  | 1.9509^***^ | 1.9672^***^ | 1.9367^***^ |
|  |  | (0.1045) |  | (0.1934) | (0.2039) | (0.1935) |
| Homemaker |  | 0.3512^***^ |  | 0.3938^*^ | 0.2703 | 0.3978^*^ |
|  |  | (0.1004) |  | (0.1939) | (0.1978) | (0.1937) |
| Student |  | -0.7626^***^ |  | -0.7402^***^ | -0.8682^***^ | -0.7328^***^ |
|  |  | (0.1058) |  | (0.1981) | (0.2042) | (0.1978) |
| Retired |  | -0.8378^***^ |  | -0.7694^***^ | -0.8350^***^ | -0.7457^***^ |
|  |  | (0.0867) |  | (0.1819) | (0.1849) | (0.1816) |
| Work Hours |  |  | -0.0225^***^ | -0.0062 | -0.0066 | -0.0048 |
|  |  |  | (0.0037) | (0.0085) | (0.0085) | (0.0084) |
| Work Hours Squared |  |  | 0.0004^***^ | 0.0002 | 0.0002 | 0.0002 |
|  |  |  | (0.0001) | (0.0001) | (0.0001) | (0.0001) |
| Interaction Terms |  |  |  |  |  |  |
| LCPU * |  |  |  |  |  |  |
| Employed Part Time |  |  |  |  | 0.2579 |  |
|  |  |  |  |  | (0.1634) |  |
| Unemployed |  |  |  |  | 0.7405^***^ |  |
|  |  |  |  |  | (0.2000) |  |
| Volunteer |  |  |  |  | -3.6624 |  |
|  |  |  |  |  | (2.6685) |  |
| Disabled |  |  |  |  | -0.0574 |  |
|  |  |  |  |  | (0.2300) |  |
| Homemaker |  |  |  |  | 0.7350^*^ |  |
|  |  |  |  |  | (0.2835) |  |
| Student |  |  |  |  | 0.7810^**^ |  |
|  |  |  |  |  | (0.2667) |  |
| Retired |  |  |  |  | 0.5161 |  |
|  |  |  |  |  | (0.2787) |  |
| Work Hours |  |  |  |  |  | -0.0106^***^ |
|  |  |  |  |  |  | (0.0030) |
| Women | 0.5611^***^ | 0.5713^***^ | 0.5955^***^ | 0.6097^***^ | 0.6113^***^ | 0.6120^***^ |
|  | (0.0439) | (0.0443) | (0.0434) | (0.0441) | (0.0442) | (0.0441) |
| Age | -0.4973^***^ | -0.4953^***^ | -0.5068^***^ | -0.5006^***^ | -0.5026^***^ | -0.5011^***^ |
|  | (0.0090) | (0.0100) | (0.0098) | (0.0109) | (0.0109) | (0.0109) |
| Race ^b^ |  |  |  |  |  |  |
| Black | -0.0077 | -0.1349 | -0.0078 | -0.1479^*^ | -0.1486^*^ | -0.1464^*^ |
|  | (0.0704) | (0.0705) | (0.0732) | (0.0727) | (0.0725) | (0.0727) |
| Native American | 0.9093^***^ | 0.7737^**^ | 0.9107^***^ | 0.7788^**^ | 0.7737^**^ | 0.7713^**^ |
|  | (0.2341) | (0.2417) | (0.2446) | (0.2524) | (0.2494) | (0.2501) |
| Hawaiian | 0.8730^*^ | 0.7521 | 0.5588 | 0.4275 | 0.4251 | 0.4268 |
|  | (0.4356) | (0.4255) | (0.4403) | (0.4321) | (0.4327) | (0.4316) |
| Asian | 0.8757^***^ | 0.8218^***^ | 0.9239^***^ | 0.8719^***^ | 0.8737^***^ | 0.8687^***^ |
|  | (0.0931) | (0.0939) | (0.0984) | (0.0993) | (0.0993) | (0.0993) |
| Multi-Racial | 0.4058^**^ | 0.3217^*^ | 0.3783^*^ | 0.3051^*^ | 0.3063^*^ | 0.3015^*^ |
|  | (0.1546) | (0.1488) | (0.1538) | (0.1474) | (0.1469) | (0.1469) |
| Hispanic | 0.4028^***^ | 0.3524^***^ | 0.3843^***^ | 0.3191^***^ | 0.3181^***^ | 0.3173^***^ |
|  | (0.0759) | (0.0746) | (0.0796) | (0.0789) | (0.0792) | (0.0790) |
| Marital Status ^c^ |  |  |  |  |  |  |
| Single | -0.4409^***^ | -0.4601^***^ | -0.4657^***^ | -0.4809^***^ | -0.4794^***^ | -0.4796^***^ |
|  | (0.0590) | (0.0606) | (0.0604) | (0.0622) | (0.0623) | (0.0623) |
| Widowed | -0.4498^***^ | -0.1379 | -0.5385^***^ | -0.1792 | -0.1675 | -0.1673 |
|  | (0.1170) | (0.1174) | (0.1207) | (0.1201) | (0.1203) | (0.1205) |
| Divorced | 0.1700^*^ | 0.0978 | 0.1558 | 0.0745 | 0.0722 | 0.0716 |
|  | (0.0857) | (0.0871) | (0.0881) | (0.0893) | (0.0891) | (0.0893) |
| Educational Attainment ^d^ |  |  |  |  |  |  |
| High School | -0.6239^***^ | -0.4694^***^ | -0.5880^***^ | -0.4590^***^ | -0.4612^***^ | -0.4636^***^ |
|  | (0.0733) | (0.0737) | (0.0768) | (0.0772) | (0.0778) | (0.0774) |
| Some College | -0.7683^***^ | -0.5456^***^ | -0.7303^***^ | -0.5444^***^ | -0.5502^***^ | -0.5532^***^ |
|  | (0.0770) | (0.0771) | (0.0788) | (0.0778) | (0.0781) | (0.0780) |
| College Degree or Higher | -1.4078^***^ | -1.1742^***^ | -1.3547^***^ | -1.1669^***^ | -1.1723^***^ | -1.1773^***^ |
|  | (0.0752) | (0.0743) | (0.0801) | (0.0786) | (0.0791) | (0.0790) |
| Family Income | -0.3244^***^ | -0.2786^***^ | -0.3195^***^ | -0.2829^***^ | -0.2827^***^ | -0.2827^***^ |
|  | (0.0126) | (0.0121) | (0.0126) | (0.0122) | (0.0122) | (0.0122) |
| Religious Attendance | -0.1280^***^ | -0.1133^***^ | -0.1232^***^ | -0.1080^***^ | -0.1080^***^ | -0.1081^***^ |
|  | (0.0141) | (0.0136) | (0.0148) | (0.0142) | (0.0142) | (0.0142) |
| Religious Salience | -0.0341^**^ | -0.0416^***^ | -0.0342^**^ | -0.0420^***^ | -0.0420^***^ | -0.0423^***^ |
|  | (0.0103) | (0.0100) | (0.0107) | (0.0104) | (0.0105) | (0.0105) |
| Mental Health Treatment | 3.9336^***^ | 3.7531^***^ | 3.9122^***^ | 3.7402^***^ | 3.7377^***^ | 3.7377^***^ |
|  | (0.0523) | (0.0536) | (0.0536) | (0.0545) | (0.0544) | (0.0545) |
| Drug Control Variables |  |  |  |  |  |  |
| Age of First Alcohol Use | 0.4111^***^ | 0.3934^***^ | 0.4029^***^ | 0.3894^***^ | 0.3866^***^ | 0.3893^***^ |
|  | (0.0523) | (0.0529) | (0.0538) | (0.0541) | (0.0539) | (0.0541) |
| Tobacco | -0.0396 | -0.0389 | -0.0428 | -0.0353 | -0.0329 | -0.0325 |
|  | (0.0249) | (0.0248) | (0.0249) | (0.0247) | (0.0248) | (0.0247) |
| Cocaine | -0.0758 | -0.1429^*^ | -0.0502 | -0.1214 | -0.1139 | -0.1190 |
|  | (0.0644) | (0.0634) | (0.0686) | (0.0674) | (0.0673) | (0.0674) |
| Stimulants | 0.4379^***^ | 0.4431^***^ | 0.4287^***^ | 0.4331^***^ | 0.4330^***^ | 0.4344^***^ |
|  | (0.0659) | (0.0657) | (0.0678) | (0.0674) | (0.0673) | (0.0674) |
| Sedatives | 0.4348^***^ | 0.3810^***^ | 0.4538^***^ | 0.4065^***^ | 0.4067^***^ | 0.4019^***^ |
|  | (0.0685) | (0.0689) | (0.0728) | (0.0726) | (0.0728) | (0.0728) |
| Tranquilizer | 0.4088^***^ | 0.4008^***^ | 0.3893^***^ | 0.3861^***^ | 0.3855^***^ | 0.3872^***^ |
|  | (0.0527) | (0.0527) | (0.0540) | (0.0541) | (0.0538) | (0.0540) |
| Inhalant | 0.4764^***^ | 0.4774^***^ | 0.4915^***^ | 0.4879^***^ | 0.4923^***^ | 0.4949^***^ |
|  | (0.0705) | (0.0708) | (0.0720) | (0.0719) | (0.0723) | (0.0722) |
| Pain Killer | 0.3876^***^ | 0.3734^***^ | 0.3991^***^ | 0.3768^***^ | 0.3744^***^ | 0.3758^***^ |
|  | (0.0567) | (0.0574) | (0.0577) | (0.0579) | (0.0583) | (0.0579) |
| Heroine | 0.6484^***^ | 0.5122^***^ | 0.6026^***^ | 0.4826^**^ | 0.4626^**^ | 0.4450^**^ |
|  | (0.1458) | (0.1454) | (0.1546) | (0.1541) | (0.1554) | (0.1549) |
| Marijuana | 0.2160^***^ | 0.1406^*^ | 0.2446^***^ | 0.1516^*^ | 0.1490^*^ | 0.1488^*^ |
|  | (0.0631) | (0.0626) | (0.0637) | (0.0630) | (0.0635) | (0.0629) |
| PCP | -0.0499 | -0.0996 | -0.0357 | -0.0798 | -0.0820 | -0.1104 |
|  | (0.1721) | (0.1676) | (0.1772) | (0.1727) | (0.1731) | (0.1721) |
| MDMA | 0.4237^***^ | 0.4998^***^ | 0.4101^***^ | 0.4819^***^ | 0.4678^***^ | 0.4921^***^ |
|  | (0.0808) | (0.0800) | (0.0830) | (0.0824) | (0.0830) | (0.0820) |
| Risky Behavior | 0.6801^***^ | 0.7073^***^ | 0.6722^***^ | 0.6988^***^ | 0.6977^***^ | 0.6985^***^ |
|  | (0.0305) | (0.0308) | (0.0318) | (0.0318) | (0.0318) | (0.0318) |
| Survey Year ^e^ |  |  |  |  |  |  |
| 2009 | -0.0971 | -0.1080 | -0.1224 | -0.1278 | -0.1306 | -0.1309 |
|  | (0.1010) | (0.1026) | (0.1034) | (0.1040) | (0.1041) | (0.1037) |
| 2010 | -0.0318 | -0.0457 | -0.0110 | -0.0228 | -0.0247 | -0.0234 |
|  | (0.1043) | (0.1049) | (0.1084) | (0.1085) | (0.1083) | (0.1081) |
| 2011 | -0.0132 | -0.0179 | -0.0000 | 0.0047 | 0.0015 | 0.0016 |
|  | (0.1030) | (0.1030) | (0.1085) | (0.1075) | (0.1076) | (0.1074) |
| 2012 | 0.0200 | 0.0043 | -0.0053 | -0.0131 | -0.0151 | -0.0161 |
|  | (0.1069) | (0.1042) | (0.1117) | (0.1090) | (0.1087) | (0.1092) |
| 2013 | 0.1322 | 0.1260 | 0.1271 | 0.1290 | 0.1274 | 0.1275 |
|  | (0.1243) | (0.1215) | (0.1263) | (0.1237) | (0.1233) | (0.1232) |
| 2014 | -0.0571 | -0.0724 | -0.0391 | -0.0458 | -0.0469 | -0.0479 |
|  | (0.0983) | (0.0976) | (0.1005) | (0.0996) | (0.0994) | (0.0996) |
| 2015 | -0.2090 | -0.2188^*^ | -0.2241^*^ | -0.2207 | -0.2178 | -0.2211 |
|  | (0.1077) | (0.1078) | (0.1129) | (0.1132) | (0.1128) | (0.1132) |
| 2016 | -0.1288 | -0.1299 | -0.1395 | -0.1280 | -0.1293 | -0.1297 |
|  | (0.1019) | (0.1019) | (0.1063) | (0.1054) | (0.1053) | (0.1055) |
| 2017 | -0.0290 | -0.0115 | -0.0282 | 0.0010 | 0.0035 | -0.0004 |
|  | (0.1070) | (0.1062) | (0.1104) | (0.1099) | (0.1093) | (0.1097) |
| 2018 | 0.2380^*^ | 0.2326^*^ | 0.2339^*^ | 0.2430^*^ | 0.2439^*^ | 0.2429^*^ |
|  | (0.1063) | (0.1057) | (0.1105) | (0.1097) | (0.1088) | (0.1094) |
| 2019 | 0.4783^***^ | 0.4912^***^ | 0.4980^***^ | 0.5262^***^ | 0.5249^***^ | 0.5243^***^ |
|  | (0.1185) | (0.1168) | (0.1196) | (0.1184) | (0.1183) | (0.1182) |
| Constant | 13.2429^***^ | 12.8485^***^ | 13.3978^***^ | 12.8359^***^ | 12.8948^***^ | 12.7944^***^ |
|  | (0.1842) | (0.1953) | (0.1949) | (0.2627) | (0.2631) | (0.2611) |
| Observations | 158313 | 158020 | 148965 | 148917 | 148917 | 148917 |
| *R*^2^ | 0.227 | 0.236 | 0.228 | 0.238 | 0.238 | 0.238 |
| Source: 2008-2019 National Survey of Drug Use and Health, n= 484,732  Standard errors in parentheses  ^*^ *p* < 0.05, ^**^ *p* < 0.01, ^***^ *p* < 0.001  ^a^ Employed Full-Time  ^b^ White, non-Hispanic serves as the reference category  ^c^ Married serves as the reference category  ^d^ Less than a high school degree serves as the reference category  ^e^ 2008 serves as the reference category | | | | | | |
